# Functional activity limitation of leprosy cases in an endemic area in Indonesia and recommendations for integrated participation program in society

**DOI:** 10.1371/journal.pntd.0010646

**Published:** 2022-08-19

**Authors:** Sri Linuwih Menaldi, Melinda Harini, Nelfidayani Nelfidayani, Yunia Irawati, Steven Setiono, Luh Karunia Wahyuni, Tri Rahayu, Gitalisa Andayani, Dewi Friska, Boya Nugraha

**Affiliations:** 1 Department of Dermatology and Venereology, Faculty of Medicine Universitas Indonesia, Dr. Cipto Mangunkusumo Hospital, Jakarta, Indonesia; 2 Department of Physical Medicine and Rehabilitation, Faculty of Medicine Universitas Indonesia, Dr. Cipto Mangunkusumo Hospital, Jakarta, Indonesia; 3 Department of Ophthalmology, Faculty of Medicine Universitas Indonesia, dr. Cipto Mangunkusumo Hospital, Jakarta, Indonesia; 4 Department of Community Medicine, Faculty of Medicine Universitas Indonesia, Jakarta, Indonesia; 5 Department of Rehabilitation Medicine, Hannover Medical School, Hannover, Germany; Hospital Infantil de Mexico Federico Gomez, MEXICO

## Abstract

**Background:**

Leprosy continues to be a health problem in Indonesia, with incidence reaching over 10,000 new cases by 2021. Leprosy-related disabilities cause limitation of patients’ activity and participation in social activities. To date, no studies have been conducted in Indonesia which investigates disability in terms of bodily function, structure impairment, limitations in performing daily activities, and restrictions in participation in social activities in leprosy patients. This study is aimed to determine the demographic and clinical characteristics that might affect functional activity limitations of leprosy patients in endemic areas in Indonesia.

**Methods and findings:**

A cross-sectional study was conducted on 267 retrospectively-diagnosed cases of leprosy. The Screening of Activity Limitation and Safety Awareness (SALSA) scale was used to measure functional activity limitation, which comprises five domains: vision, mobility, self-care, work with hands, and dexterity. Differences among variables were evaluated using Kruskal-Wallis and Mann-Whitney test. The mean age of participants was 51.89±13.66 years, the majority of which were men (62.5%), uneducated (48.3%), and classified as type 2 in the World Health Organization (WHO) disability grading for hands and feet (66.3% and 68.2%, respectively). Assessment using the SALSA Scale showed 28.5% of subjects were without limitation, 43.8% with mild limitation, 13.5% with moderate limitation, 9.4% with severe limitation, and 4.9% with extreme limitation. Significant differences in the total SALSA Scale were found between age groups (p = 0.014), educational level (p = 0.005), occupation (p<0.001), and WHO disability grades (p<0.001). Multivariate analysis showed that the most significant factor influencing the total score of SALSA was disability grading for feet (score = 0.31, p <0.001) followed by occupational status, disability grading for eyes, and age. Limitation of functional activity was significantly correlated to becoming unemployed with the odds 2.59.

**Conclusion:**

People affected by leprosy are prone to have functional activity limitation, especially the elderly, uneducated, unemployed and those with multiple disabilities. If they can overcome their barriers in functional activities, they will have better occupational opportunities.

## Author summary

Efforts to achieve elimination are mainly focused on the curative or treatment aspects. However, it is important to be aware that disability due to leprosy still occurs even after treatment has been completed. The WHO definition for disability includes bodily function and structure impairment, activity limitation and participation restriction. In leprosy, disability can cause broad problems and have an impact on education, employment, and lead to social and economic problems. This study is aimed to obtain data on activity limitation in people affected by leprosy in a district with high prevalence and a leprosy community. A total of 267 patients participated in this study. Physical examination was performed on the eye, hand and foot disabilities based on WHO guidelines, and patients were asked to complete the SALSA (The Screening Activity Limitation Safety Awareness) questionnaire to measure activity limitations. Based on the total SALSA score, this study revealed that 71.5% of participants experienced activity limitations. Occupational status indicates the ability of people affected by leprosy to optimize their functional activities. Thus, integrated management of disability is essential to improve activity limitations.

## Introduction

Leprosy is considered as an ancient infectious diseases which has spread all around the world since 2000 BC [1]. The reduction in the number of new cases has been gradual and uniform for the past 10 years globally [2]. Nowadays, some countries have eradicated/eliminated leprosy, however in other countries including Indonesia, leprosy incidence reaches more than 10,000 new cases. According to 2010 and 2019 data, leprosy incidence in Indonesia ranges from 17,012 to 20,023. This leads to Indonesia ranking third in terms of countries with the most leprosy cases in the world [2].

In reality, Indonesia has given great effort in leprosy management and reached leprosy elimination status in 2000 [1], as defined by leprosy prevalence less than 1 per 10,000 population [1]. The prevalence and incidence of leprosy has decreased even further throughout the years. Until 2017, leprosy prevalence in Indonesia reached 0.7 cases per 10,000 population, with 6.08 new cases per 100,000 population. Nevertheless, there are still 10 provinces in Indonesia that have not achieved elimination, making Indonesia an endemic country of leprosy. There is still a great amount of work to be done, in order to eliminate leprosy completely in Indonesia. [1].

Global attention to eliminating leprosy has focused on curative efforts, including Indonesia.[1,2]. Additionally, leprosy is well known as one of the most frequent causes of disability, even years after the patient is cured [3]. People affected by leprosy are prone to have physical disability grade progression with a probability of 35% [3].

According to WHO, disability is an umbrella definition for impairment of bodily function and structure, activity limitation, and participation restriction [4]. To the best of our knowledge, there are no studies in Indonesia integrating those three aspects. This study aimed to determine the impairment in bodily function and structure, as well as functional activity limitation of leprosy patients living in endemic areas in Indonesia.

## Materials and methods

### Ethical aspects

The study protocol was approved by the Ethics Committee of Faculty of Medicine, Universitas Indonesia under ethical clearance No. 0310/UN2.F1/ETIK/2018 and No. 698/UN2.F1/ETIK/PPM.00.02/2019, and was designed in consideration of the principles proposed by Helsinki Declaration. Written formal consent was obtained directly from participants. For child participants, the written formal consent was obtained from the parent/guardian.

### Study design, location and period

A cross-sectional study was done at Singkawang, West Kalimantan and on a leprosy community area at Tangerang, Banten, Indonesia. As Indonesia is an endemic country, both of the provinces were classified as provinces with high prevalence of leprosy. The difference between the two populations lied in patients’ residences, in which leprosy patients at Singkawang lived in their own houses and its surrounding areas, while leprosy patients in Tangerang lived within a leprosy community, called Sitanala Village. Data collection was performed in April 2018 and July 2019. The study was conducted by the KATAMATAKU team, a multidisciplinary (expert) group consisting of ophthalmologists, dermatologists, and physiatrists from Cipto Mangunkusumo General Hospital and the Faculty of Medicine Universitas Indonesia.

### Population or sample: Inclusion and exclusion criteria

Participants were all leprosy patients who lived in a district of high leprosy prevalence (Singkawang, West Kalimantan) and in a leprosy community (Sitanala Village, Tangerang). Inclusion criteria were leprosy patients who have given written consent to participate in documented research. Each participant completed the SALSA questionnaire and underwent all examination series which included eye, skin, and extremities examination. Participants under 18 years old required formal consent (written) from their parents or legal guardians. The exclusion criteria were patients who were still on leprosy medication based on the patients’ self-report, and patients with incomplete data.

### Study protocol

Data were collected by interviewing and examining a total of 267 patients in Singkawang and Tangerang. Closed-question questionnaires were administered as face-to-face interviews for all subjects who consented to participate in the study. Basic demographic data including age, gender, marital status, educational background, and occupational status were obtained.

### Assessment tools

#### Physical examinations

The physical examinations performed in this study included the detection of leprosy signs and symptoms such as abnormalities and deformities in eyes (uncorrected and best corrected visual acuity using Snellen chart), skin, and extremities (inspection of lesion and deformity, motoric and sensory nerve function test).

#### Disability grading

Disability grades were assessed using the WHO disability grading system which classified leprosy patients according to disabilities of the eyes, hands, and feet [5]. The highest score for eyes, hands, or feet is used as an indicator of severity of impairment bodily structure and function [6].

#### The Screening Activity Limitation Safety Awareness (SALSA)

The SALSA scale was used to measure the activity limitation component of disability. The scale is a cross cultural tool, comprising 20 items of daily activities related to the three domains of mobility, self-care and work [7]. The questionnaire is completed through interview, and it is a subjective in nature, placing the interviewee at the center by presenting how the participants perceive it at a functional level. For this study, we used the Indonesian version of SALSA. The scale was designed to be applied for people with peripheral neuropathy. The score ranges from 0 to 80, with the following recommended cutoffs/categories: no significant limitation (0–24), mild limitation (25–39), moderate limitation (40–49), severe limitation (50–59) and extreme limitation (60–80) [7,8].

#### Occupational status

Classification of workers into formal and informal sector workers were not easy to define, since there is no international consensus about the definition of the two sectors. Managerial workers in Indonesia are considered to represent formal sector workers, consisting of professionals, technicians, leadership and management personnel, administrative personnel, sales workers, and service workers. Formal work refers to work in which a company hires an employee under an established working agreement that include, salary or wages, health benefits, and defined work hours and workdays. In most instances, employees do not work under a signed contract, but rather work under the agreement reached when the employer offered the job to the employee. This agreement remains in force until the employer makes a change and informs an employee about such changes. Employees in a formal work agreement are often given an annual performance evaluation and are eligible for salary increases and promotions based on their performance. Formal work tends to require a higher level of education or training compared to informal work. Informal worker refers to workers in jobs that rely on physical strength, and in business groups in Indonesia, are usually included in the types of jobs in the agricultural, forestry, hunting, fishery, production personnel, transportation and rough workers [9,10].

#### Analysis of results and statistics

Data was stored and organized in an electronic spreadsheet. Statistical analysis was performed using SPSS software, 20.0 version. The SALSA scoring system was used to determine the effect of activity limitation based on subjects’ occupation. Descriptive statistics of mean, median, and standard deviation were used to summarize data while comparisons among variables were evaluated using Kruskal-Wallis and Mann Whitney tests. Multivariate analysis was performed to identify factors affecting functional activities and Pearson chi-square was performed to evaluate correlation between functional activity and occupational status. Alpha level was set at *p*< 0.05.

## Results

A total of 359 people were invited to participate in this study. However, only 267 participants met the inclusion criteria and were included in the analysis. Table 1 shows the demographic characteristics of the participants in this study. Mean age was 51.89±13.66 years old. The majority of participants were male, with a male-to-female ratio of 1.7:1. Most of the participants were uneducated (48.3%) and attained only primary education (40.1%). Around half of them (53.2%) have informal occupation.

**Table 1:** Demographic characteristics of study participants.

Variables	Total subjects (n = 267)	
	n	%
**Age (mean ± SD)**	**51.89 ± 13.66**	
< 18	6	2.2
18–30	9	3.4
31–45	72	27
46–60	108	40.4
>60	72	27
**Gender**		
Males	167	62.5
Females	100	37.5
**Educational Background** *		
Uneducated	129	48.3
Primary education	107	40.1
Secondary education	28	10.5
Tertiary education	3	1.1
**Occupational status** **		
Formal	1	0.4
Informal	142	53.2
Unemployment	52	19.5
Unemployed	72	27

*Uneducated: no education; primary education: elementary, junior high school; secondary education: senior high school; tertiary education: undergraduate

**Formal: teacher; Informal: construction worker, driver, farmer, freelancer, maid; unemployment: not working; unemployed: student, housewife, child

The subjects were mostly in grade 2 for hands 66.3% (n = 177) and feet 68.2% (n = 182) disability according to the WHO disability grading for extremities. Meanwhile, in terms of eye disability, the majority of subjects were in grade 0 (n = 222, 83.1%). The median (min-max) SALSA score was 30 (10–77). Most of the patients (n = 191, 71.5%) had activity limitations; 117 (43.8%) had mild limitation, 36 (13.5%) had moderate limitation, 25 (9.4%) had severe limitation, and 13 (4.9%) had extreme limitation.

Table 2 shows several factors which were compared with functional activity limitation evaluated using the SALSA Scale. Significant differences were observed between age (p = 0.014), educational backgrounds (p = 0.005), occupation (p < 0.001), and WHO disability grading for eyes, hand and feet (p < 0.001). Comparison of SALSA scores between age groups showed that the younger age group did not have significant activity limitations (median 24) with a significant difference between the <18 years (p = 0.036) and 18–30 years (p = 0.022) age groups compared to the 31–45 years age group as reference.

**Table 2:** Comparison of SALSA Score among demographic and clinical characteristics.

Variables	Median (min-max)					
	Total Score SALSA	Domain SALSA				
		Vision (eyes)	Mobility (feet)	Self-care	Work (hands)	Dexterity (hands)
**Age (years old)**						
< 18	24 (20–29)	1 (1–2)	4.5 (4–7)	3 (3–6)	7.5 (7–13)	5 (5–8)
18–30	24 (19–37)	1 (1–2)	5 (4–16)	3 (2–3)	9 (6–13)	5 (5–10)
31–45	30 (19–67)	2 (1–4)	8 (3–16)	3 (3–10)	7 (6–28)	8 (4–19)
46–60	31 (19–73)	2 (1–4)	8 (3–16)	3 (3–12)	7 (6–27)	8 (4–20)
>60	33 (20–77)	2 (0–4)	7 (4–16)	3 (2–10)	9 (6–28)	8 (5–20)
p^**^	***0*.*014***	*0*.*107*	*0*.*084*	***0*.*030***	*0*.*103*	***0*.*002***
**Gender**						
Males	30 (19–77)	1 (1–4)	7 (4–16)	3 (2–12)	8 (6–28)	8 (4–20)
Females	29.5 (19–73)	1 (1–3)	8 (3–16)	3 (2–10)	8 (6–28)	7.5 (4–20)
p^*^	*0*.*578*	*0*.*035*	*0*.*398*	*0*.*208*	*0*.*816*	*0*.*211*
**Educational Background**						
Uneducated	32 (19–77)	1 (1–4)	8 (3–16)	4 (2–11)	9 (6–28)	8 (4–20)
Primary education	29 (20–73)	1 (1–4)	7 (3–16)	3 (2–12)	7 (6–28)	8 (5–20)
Secondary education	28 (19–54)	1.5 (1–4)	7 (4–14)	3 (3–10)	7 (6–22)	7.5 (5–19)
Tertiary education	21 (19–23)	1 (1–2)	5 (4–6)	3 (3–3)	7 (6–7)	5 (5–5)
p^**^	***0*.*005***	*0*.*334*	*0*.*193*	***0*.*044***	***0*.*013***	***0*.*028***
**Occupational Status**						
Formal	19	1	4	3	6	5
Informal	28 (19–73)	1 (1–4)	7 (3–16)	3 (3–12)	7 (6–27)	8 (4–20)
Unemployment	36.5 (20–77)	2 (1–4)	9 (4–16)	5 (2–11)	11 (6–28)	9 (5–20)
Unemployed	31.5 (19–67)	1 (1–3)	9 (3–16)	3 (3–10)	9.5 (6–28)	8 (4–19)
p^**^	***<0*.*001***	*0*.*189*	***0*.*004***	*0*.*159*	***<0*.*001***	***0*.*036***
**WHO Disability grading for feet**						
Grade 0	23 (19–49)	1 (1–3)	5 (4–14)	3 (3–6)	7 (6–19)	5 (5–19)
Grade 1	24 (19–58)	2 (1–4)	5 (3–15)	3 (3–8)	7 (6–15)	7 (4–20)
Grade 2	34 (19–77)	1 (1–4)	9 (3–16)	4 (2–12)	9 (6–28)	10 (4–20)
p^**^	***<0*.*001***	*0*.*197*	***<0*.*001***	***<0*.*001***	***<0*.*001***	***<0*.*001***
**WHO Disability grading for hands**						
Grade 0	23 (19–64)	1 (1–4)	5.5 (4–14)	3 (3–12)	7 (6–25)	5 (4–19)
Grade 1	26.5 (19–59)	2 (1–3)	6.5 (3–16)	3 (2–9)	7.5 (7–21)	6 (5–18)
Grade 2	34 (19–77)	2 (1–4)	9 (3–16)	4 (2–11)	10 (6–28)	10 (4–20)
p^**^	***<0*.*001***	***0*.*005***	***<0*.*001***	***<0*.*001***	***<0*.*001***	***<0*.*001***
**WHO Disability grading for eyes**						
Grade 0	29 (19–77)	1 (1–4)	7 (3–16)	3 (2–12)	7 (6–28)	8 (4–20)
Grade 1	28.5 (20–59)	1.5 (1–3)	8.5 (4–13)	3 (3–9)	9.5 (7–21)	6.5 (5–18)
Grade 2	41 (22–73)	2 (1–4)	9 (4–15)	6 (3–10)	11 (6–28)	11 (5–20)
p^**^	***<0*.*001***	***<0*.*001***	***0*.*042***	***0*.*006***	***<0*.*001***	***<0*.*001***

Note

*Mann-Whitney test

**Kruskal Wallis test

Multivariate analysis using linear regression revealed that the total score SALSA was significantly affected by disability grading for feet, occupational status, disability grading for eyes, and age (p-value of the overall regression model <0.001 with R^2^ 0.243). Among these overall factors, disability grading for feet was the factor with greatest influence on the total SALSA score (score = 0.31 with p value <0.001) followed by occupational status, disability grading for eyes, and age (Table 3). Multivariate analysis also revealed that disability grading for hands and educational background did not affect the total SALSA score.

**Table 3:** Multivariate Analysis of Factors Affecting Total Score SALSA.

	*Unstandardized coefficients* (B)	*Standardized coefficients* (β)	CI 95%	p value*	
WHO Disability grading for feet	4.86	0.31	3.09–6.64	<0.001	<0.001
Occupational Status	3.09	0.21	1.49–4.69	<0.001	
WHO Disability grading for eyes	3.44	0.19	1.48–5.39	0.001	
Age	0.15	0.16	0.05–0.26	0.004	

^*^Linear regression model, significancy p value <0.05

Table 4 described the correlation between functional activity limitation and occupational status. Functional activity was statistically correlated with occupational status (p value = 0.001). Based on the results shown in Table 4, the odds ratio (OR) was 2.59, indicating that people who have limited functional activities have a 2.59 times greater chance of becoming unemployed compared to people who do not have functional activity limitations.

**Table 4:** Correlation between Functional Activity and Occupation Status.

	Unemployed (n)	Employed (n)	p value
Functional Activity Limitations	101	90	0,001*
Independent Functional Activity	23	53	

^*^Pearson chi-square test, significant if p<0.05

## Discussion

Leprosy-related disability is a challenge, especially in Indonesia. According to the International Classification of Function, Disability and Health (ICF) WHO in 2001, disability can be defined as a difficulty pertaining to bodily structure and function, activity limitations and participation restrictions. It also recognizes the role of physical and social environment factors in how the patients overcome the barriers from their disabilities [11].

This study determined several factors that affect the functional activity limitations of leprosy patients in endemic areas in Indonesia, and the relationship to the type of occupation as a mean of participation in society. The population of this study consisted mostly of male (62.5%) subjects aged 45–60 years (40.4%), uneducated (48.3%) and informal occupation (53.2%). These findings showed that most of our subjects have low level of education and low occupational status. They had the same context of socioeconomic vulnerability. We compared our data with studies from two other countries with the highest leprosy cases in the world, namely Brazil and India. The mean age in our study was 51.89 ± 13.66, in line with previous studies [3,12,13]. In 2012, a study of five leprosy endemic areas in Indonesia reported that the median age of people affected by leprosy in Indonesia was 42.5 years old [12]. Data from Brazil reported that from 2000 to 2017, people affected by leprosy were mostly in the 30–59 years old age group (66.3%) [3]. Another study in 2016–2017 found that the mean (SD) age of leprosy-affected people in four leprosy endemic states of India was 39 years old [13,14].

In this study, male participants were dominant compared to female with a 1.7:1 ratio, similar to the ratio reported in a 2012 Indonesian study [12]. Compared to other countries, the male prevalence is higher than the proportion in Brazil (1.1:1) but almost similar to the proportion in India (1.8:1) [13,14]. Noordeen [14] analyzed that due to differences in the sociocultural behavior between the genders and the lifestyle of men who tend to expose themselves to greater risks of infection, as well as biological and economic factors, the male population may well be related to the greater detection of leprosy cases.

Most of subjects are uneducated (48.3%) or have attained only primary education (40.1%). This is similar to the 2012 Indonesian report which showed comparable percentages of illiterate subjects (31.9%) and subjects who have attained only primary education (43.9%) [12]. In Indonesia, primary educational level consists of 6 years of elementary school and 3 years of junior high school. Leprosy-affected people in India also has similar educational level distribution; specifically 0 year (21%), 1–5 years (21%), and 6–10 years (38%) [13].

Concerning occupational status, most participants have informal occupation (53.2%). In India, the occupational distributions of leprosy-affected people were also dominated by informal sector, such as agriculture (33%) and labor (30%) [13]. The Brazilian study did not describe the occupational distribution clearly [14]. The limitations in participation may be caused by several factors including self-stigmatization, activity limitation, family related issues, poverty, and low level of education as well as the community’s tendency to stigmatize these patients, due to a fear of contagion. Moreover, the problem is also likely to be related to insufficient rehabilitation services including community-based rehabilitation (CBR) programs. CBR programs along with other disability prevention strategies were found to improve social participation. Integrating CBR in existing vertical leprosy programs and monitoring using WHO CBR indicators could be implemented. New case diagnosis and management may be systematically linked to CBR programs, therefore, it should be integrated into CBR programs, as CBR services are available in Indonesia [15].

For the first time in Indonesia, this study assessed the functional activity limitation and participation restrictions of leprosy patients using internationally validated scales. In the present study, almost three-quarters of the participants (71.5%) experienced functional activity limitations, the majority of which was mild limitation in activities. This proportion is similar to the study by Santos *et al* in Brazil and Abdela *et al* in Ethiopia, which respectively found 75.9% and 71.8% of participants with activity limitations, mostly with mild activity limitations [16,17].

In our study, age groups, educational level, occupation and WHO disability grading (WHO-DG) were found to have significant effect on functional activity. Comparison of SALSA scores between age groups showed that the younger age group tend to have no significant activity limitations (median 24) with a significant difference between the <18 years age group (p = 0.036) and 18–30 years (p = 0.022), using the 31–45 years age group as reference. Older persons affected by leprosy suffered more limitation in activities compared to younger persons; this was consistent with the findings of the study conducted in Brazil [18]. The result of this study showed that age had a direct effect on activity limitations, and it was statistically significant. Age is an important factor of functional disability and self-care. In leprosy, self-care is the most important type of self-management where leprosy patients need to change their behavior to adapt to the irreversible impairment due to the disease [19]. This is especially significant among leprosy-affected people that has the potential to acquire physical disability grade progression years after being released from treatment [20]. Disability progression might also explain why leprosy-affected elderly shows lower SALSA score in the self-care sub-domain, when compared to the younger patients. Lower self-care score could be also caused by cognitive deficits that are generally found in elderly [21].

This study found that subjects with lower educational levels had worse SALSA scores compared to subjects with higher educational levels (p = 0.005) in the self-care, works with hands, and dexterity domains. Dexterity refers to the ability to perform skillful fine motor function of the hand, mostly done by the dominant hand. A study on leprosy patients clearly showed that if a unilaterally dominant hand was affected, the patient would be less adept at performing basic daily activities than a unilaterally affected non-dominant hand [22].

Education level affects treatment adherence in leprosy patients. Low level of education causes leprosy patients to take medicine irregularly, worsening the disability [23]. Low education level may also cause leprosy patients to not perform proper wound care to prevent leprosy complications, further worsening the defect [24]. Patients with higher educational levels may be more knowledgeable about leprosy symptoms and could have better health seeking behavior. This might result in early diagnosis of leprosy, thereby reducing disability and other related problems. Efforts on physical disability prevention and management could improve physical activities and social engagement of leprosy patients.

In this study, the subject’s occupation is one of their social participation forms. As shown in Table 4, subjects with more activity limitations were likely to have no occupation. The vicious cycle of level of disability and social participation might be mediated by stigma and discrimination faced by leprosy-affected people. The consequences of stigma can be seen from psychosocial dysfunction to isolation, rejection and participation restriction. Social destruction due to several conditions including stigma, discrimination, poverty, disability and loss of freedom are still major obstacles to be overcome by people affected by leprosy, as well as health professionals and health programs. There are three kinds of stigma related to leprosy, namely experienced stigma, perceived stigma, and self-stigma. Experienced stigma is the stigma that a person obtains from society, such as being ‘relieved’ from work or school, being divorce, denied access to public transportation, as well as discrimination. Self-stigma is a person’s feelings towards themselves that keeps them away from society. Perceived stigma is the perception, expectation, fear, or concern of discrimination and awareness of negative attitudes that society will impose on them, due to a certain condition [25].

Perceived stigma will cause people who suffer from leprosy to lose their productivity. In addition, perceived stigma can impoverish them, to the point of begging in order to survive. Stigma was the main determinant of social participation, and therefore disability. Factors related to perceived stigma were educational level, perception of knowledge about leprosy, level of disability, and cultural values [25].

Disability in this study was low, as seen in WHO disability grading for eyes, with Grade 0 being more prevalent (83.1%). Howveer, disability in hand and feet were categorized as severe, with grade 2 disability found in 66.3% and 68.2% of subjects, respectively. Globally, in 2019, the proportion of grade 2 disability cases was 5.3% among new cases. A review of new case detection in the three most highly endemic countries indicated that the number of new cases and the proportions of cases with disability have been more or less unchanged in Brazil and Indonesia for the past 5 years [2]. Preventing disability is one of the priorities of the Global Leprosy Strategy. The emphasis of the WHO strategy is early detection to prevent cases with grade 2 disability at the time of diagnosis [4]. Interestingly, even with severe disability, most of the subjects in the present study had mild limitations in activity (43.8%). This is in line with the study in Brazil which found that more leprosy-affected people had no activity limitation (40.9%) even though most of them had severe impairment with WHO disability grade 2 in 48.7% [3].

Comparison of SALSA scores between disability levels, as well as the WHO-DG for feet, hands, and eyes, showed significant differences (p<0.001) in almost all domains. The findings suggest that individuals with physical disabilities may have limitations in activities. The result of this study was consistent with a study by De Souza, *et al*. (2016) [26] which stated that SALSA scores were associated with the degree of impairment (p < 0.01), where high SALSA scores are associated with higher impairment.

Ocular complications in leprosy can occur before, during, or after treatment using the multi-drug therapy (MDT) treatment. Vision impairment in leprosy patients may result from complications of the disease or common eye problems. The most common ocular manifestations in leprosy are: lagophthalmos, entropion, and cataract [27,28]. The majority of impaired vision and blindness resulting from leprosy are preventable and curable.

Age is the most important factor for predicting the increase of visual impairment and decline in ability to perform daily living activities. Furthermore, the older the patient, the longer the duration of leprosy, and older patients have a greater possibility of developing visual impairment and blindness. In this study, disability of the hands and feet were more prevalent than the eyes because most subjects were in the age group of 40–60 years. In previous studies, individuals older than 65 years showed more visual impairment than any other age group, and persons older than 70 years tend to require assistance in daily activities. Another study found a significant increase in the prevalence of visual impairment in normal population aged 75 years and above [29,30].

Vision plays the most prominent role in performing daily activities, as 90% of information is transmitted to the brain through vision. Although the subjects in the present study had hands (Grade 2, 66.3%) and feet (Grade 2, 68.2%) disabilities, no significant disability of the eyes were observed (Grade 0, 83.1%). Even in individuals with impaired extremities, as long as they retain good vision, they can still perform daily activities due to compensation mechanisms (mild limitation with SALSA Scale, 43.8%). It is important to ensure that leprosy patients maintain a good functional vision with periodic eye examinations, at least twice a year to prevent sight-threatening complications and provide early management. Patients with ocular disabilities will have ’double handicap’ along with disabilities of the feet and hand. A previous study found that participants with severe visual impairment (20/200 or less) have three times higher odds of experiencing limitations in mobility and activity of daily living, compared to persons with visual acuity of 20/40 or better [29].

In this study, we did not find any significant srelationship between feet disability and vision, which could be explained by the fact that walking or standing more dominantly use the peripheral visual field, whereas hand and eye activities principally use the central visual field. Furthermore, the process of walking, according to behavioral studies, has a link between movement and cognition, such as memory, attention, and perceptual processes [31]. Hence, visual impairment may not be the only factor influencing movement by feet, including walking. Lastly, the leprosy patients with feet disability may still be mobile, using simple walking aids such as crutch, wheelchair, or even prosthetics.

Recognizing that disability is a major problem that requires interdisciplinary management, a multidisciplinary team was formed and named KATAMATAKU. This team aspires to collaborate and help leprosy patients to overcome their disabilities, hoping to make a difference, and to assist in government programs for leprosy management. KATAMATAKU is an Indonesian acronym for *Identifi**ka**si* *Ta**nda-Tanda* *Ma**ta*, *Ekstremi**ta**s dan* *Ku**lit pada* *Ku**sta*, or Identification of the Ocular, Extremities and Dermatological Signs in Leprosy. The main team consists of dermatologists, ophthalmologists and physiatrists from the Faculty of Medicine of Universitas Indonesia and Cipto Mangunkusumo General Hospital. This collaborative team had visited a number of endemic areas around Indonesia and met leprosy patients, mostly survivors. Community engagement and charity have become the main program of KATAMATAKU. The goals are to prevent and help people affected by leprosy overcome barriers and be included in the community so that they could have better quality of life.

Although our study has provided essential information on the profile and disabilities of leprosy patients in Indonesia, we acknowledge its limitation, which is the lack of complete data related to the patients history of medication adherence, as well as data on patient care and specialized medical attention related to prevention of disability.

## Conclusion

This study concluded that functional activity limitation is common among people affected by leprosy, with larger proportions in patients who are elderly, uneducated, unemployed, and disabled. As functional activities would be related to occupational opportunities for people affected by leprosy, integrated programs related to disability prevention and management should be carried out continuously and extensively.
